# Clinicopathological characteristics and prognosis of hepatitis B associated membranous nephropathy and idiopathic membranous nephropathy complicated with hepatitis B virus infection

**DOI:** 10.1038/s41598-021-98010-y

**Published:** 2021-09-15

**Authors:** Ruiqiang Wang, Yunqi Wu, Bowen Zheng, Xiaofeng Zhang, Dongyue An, Ningning Guo, Jin Wang, Yuanyuan Guo, Lin Tang

**Affiliations:** 1 Department of Nephrology, The First Affiliated Hospital of Zhengzhou University, Zhengzhou, 450052 Henan Province China; 2 Hebei Medical University First Affiliated Hospital, Shijiazhuang, 050030 Hebei China

**Keywords:** Nephrology, Kidney, Kidney diseases

## Abstract

The main objective of this study is to analyze the clinical and pathological features and prognosis of patients with Hepatitis B associated membranous nephropathy (HBV-MN) and idiopathic membranous nephropathy (IMN) complicated with hepatitis B virus (HBV) infection. This study will provide more basis for diagnosis and prognosis evaluation. A total of 50 patients with HBV-MN were included in this study. 56 IMN patients complicated with HBV infection diagnosed during the same period formed the control group. Parameters including blood routine, urine routine and plasma levels of albumin (ALB), serum creatinine (SCR), blood urea nitrogen (BUN), urea acid (UA), total cholesterol (T-CHO), triglycerides (TG), complement C3 and C4, glutamic pyruvic transaminase (ALT), glutamic pyruvic transaminase (AST), 24-h urinary protein quantification (24 h-TP), renal phospholipase A2 receptor (PLA_2_R) and HBV related markers during the hospitalization and outpatient follow-up study period were collected for all the patients. The proportion of male patients was high in both groups. The average age of the HBV-MN group was 37.2 ± 14.187 years old, it was younger compared with the IMN group (*P* = 0.003). Nephrotic syndrome was the major clinical manifestation among patients. There was no significant difference between the two groups in the levels of anemia, microscopic hematuria, renal dysfunction, liver dysfunction, liver cirrhosis. The level of serum C3 and C4 in the HBV-MN group was lower compared with the IMN group (*P* = 0.002, *P* = 0.014). In the HBV-MN group, serum HBV markers were negative in 6 (12%) patients, 4 patients (8%) were positive for PLA_2_R in serum, and 5 patients (10%) were positive for PLA_2_R in renal tissue. Stronger IgG1 and C1q and weaker IgG4 staining were found in HBV-MN group renal tissues (*P* = 0.003, *P* = 0.025, and *P* = 0.001, respectively). There were no statistical differences compared with serum and renal PLA_2_R between HBV-MN and IMN groups (*P* = 0.098, *P* = 0.109). During the 1-year follow-up, there was no significant difference in complete remission rate between the two groups (*P* = 0.7739). Renal biopsy is crucial to diagnose HBV-MN. IgG subtypes in the HBV-MN group were mainly IgG1 deposition, while those in IMN complicated with HBV infection group were mainly IgG4 deposition. When HBV-associated antigen and PLA_2_R are present in renal tissue, lower level of serum C3 and C4, high intensity of renal C1q and IgG1 is more supportive of HBV-MN. The positive of PLA_2_R in serum and renal tissue in differentiating HBV from IMN complicated with HBV infection remains to be discussed.

## Introduction

Hepatitis B virus (HBV) infection is prevalent worldwide, but the epidemic intensity of HBV infection varies greatly in different regions. According to the World Health Organization (WHO), there are about 257 million chronic HBV infections worldwide, with Africa and the western Pacific accounting for 68%. It is estimated that at present, the prevalence rate of HBV among the general population in China is 5%-6%, and there are about 70 million cases of chronic HBV infection^1,2^. Patients with acute and chronic hepatitis complicated with hepatitis B virus infection have a variety of extrahepatic manifestations. When organs other than the liver are affected, patients may have extrahepatic manifestations, of which 3–5% may develop kidney disease, and the most common histological type is HBV-MN^3^. At present, there is no unified diagnostic standard for HBV-MN in the world. In order to improve the accuracy of diagnosis, we retrospectively analyzed the clinical and pathological features and prognosis of 50 cases of HBV-MN and 56 cases of IMN complicated with HBV infection.

## Results

### Clinical characteristics of the participants

There were 50 patients in the HBV-MN group, including 38 (76%) males and 12 (24%) females. The mean age of the patients was 37.2 ± 14.187 years (ranging from 15 to 74 years). 56 patients diagnosed with IMN complicated with HBV infection during the same period were included in the control group. In all, 38 (67.9%) were males and 18 (32.1%) were females, average age 44.1 ± 11.874 years (range 19–68 years). The proportion of male patients was high in both groups. The age of patients in the HBV-MN group was significantly lower than in the IMN group (*P* < 0.05) (Table 1).

**Table 1:** Demographics and clinical characteristic in HBV-MN and IMN patients.

Items	HBV-MN (n = 50)	IMN (n = 56)	*P*
Gender ratio (%) (M/F)	38 (76%)/12 (24%)	38 (67.9%)/18 (32.1%)	0.353
Age (years)	37.2 ± 14.187	44.1 ± 11.874	0.003
Mean course (m)	6.53 ± 15.823	8.35 ± 24.05	0.651
Nephrotic syndrome (n, %)	48 (96%)	49 (87.5%)	0.117
Hypertension (n, %)	19 (38%)	22 (39.3%)	0.892
Anemia (n, %)	14 (28%)	9 (16.1%)	0.137
Microhematuria (n, %)	28 (56%)	26 (46.4%)	0.325
Renal failure (n, %)	3 (6%)	2 (3.6%)	0.556
Hepatic injury (n, %)	20 (40%)	18 (32.1%)	0.400
Liver cirrhosis (n, %)	3 (6%)	4 (7.1%)	0.813

Nephrotic syndrome was the main manifestation in both groups. Among them, 48 cases (96%) in the HBV-MN group, 49 cases (87.5%) in the IMN complicated with HBV infection group, and the rest showed glomerulonephritis syndrome. However, in our study, there were no significant differences in hypertension, anemia, microhematuria, renal failure, liver injury and liver cirrhosis between the two groups (Table 1).

### Serologic and clinical findings

Compared with the IMN complicated with the HBV infection group, the serum C3 and C4 levels in the HBV-MN group were lower (*P* < 0.05). However, there was no significant difference in ALB, SCR, BNU, UA, ALT, AST, T-CHO, 24 h-TP and PLA_2_R positive rate between the two groups (Table 2).

**Table 2:** Biochemical indexes of HBV-MN and IMN.

Clinical Index	HBV-MN (n = 50)	IMN (n = 56)	*P*
BUN (mmol/L)	4.99 ± 0.205	5.52 ± 0.315	0.560
SCR (µmol/L)	70.88 ± 2.710	73.71 ± 4.829	0.599
UA (µmol/L)	337.86 ± 12.555	307.27 ± 10.595	0.064
ALT (U/L)	39.90 ± 4.344	46.16 ± 7.255	0.742
AST (U/L)	41.22 ± 3.658	49.32 ± 12.269	0.192
ALB (g/L)	24.36 ± 0.853	25.31 ± 0.939	0.458
T-CHO (mmol/L)	7.22 ± 0.358	7.25 ± 0.313	0.740
24 h-TP(g)	6.05 ± 0.455	5.84 ± 0.544	0.514
C3 (g/L)	1.01 ± 0.032	1.17 ± 0.039	0.002
C4 (g/L)	0.23 ± 0.013	0.44 ± 0.161	0.014
PLA_2_R ( +) (n, %)	4 (8%)	12 (21.4%)	0.098

### Comparison of IgG and complements depositions intensity

**Figure 1:**
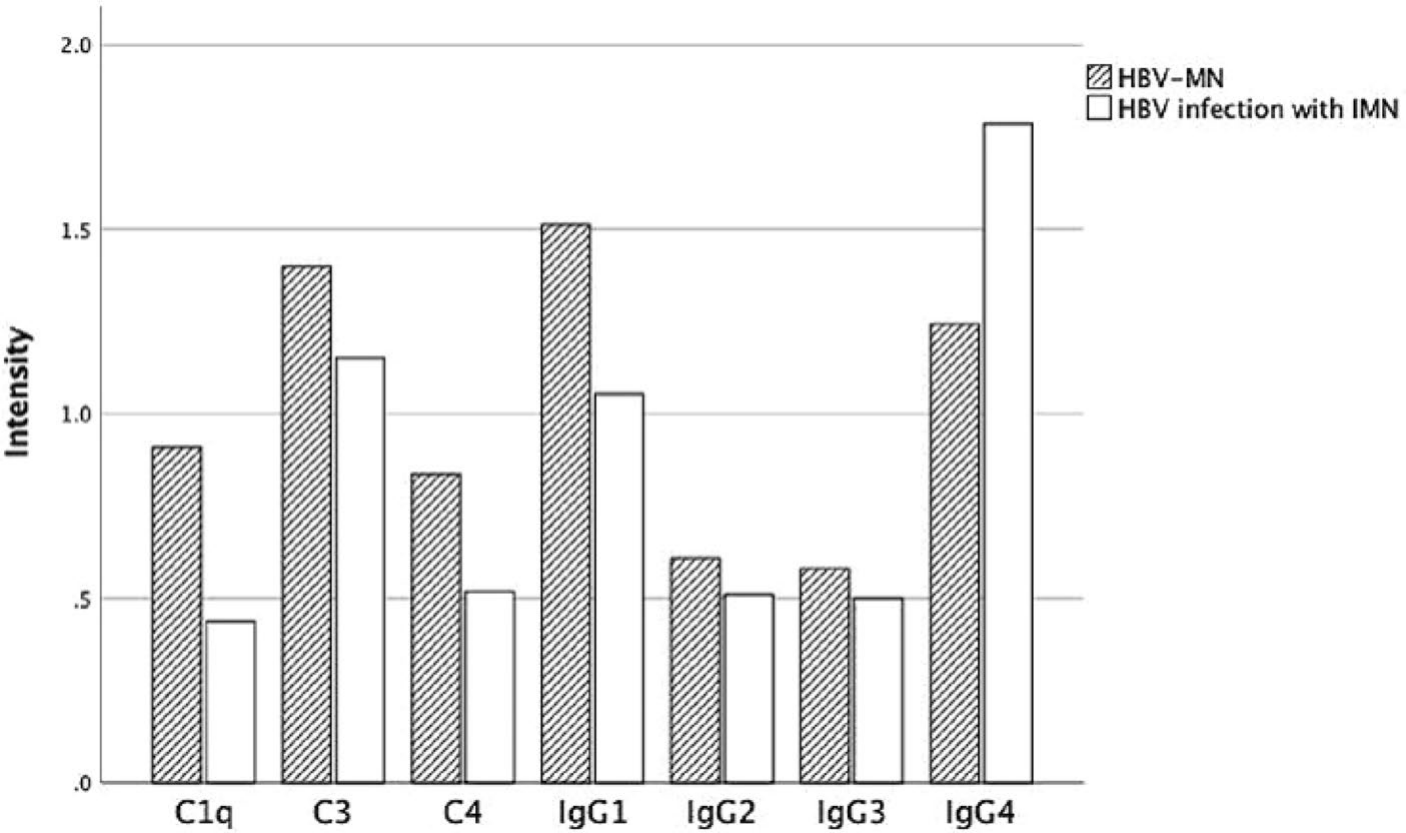
The deposition intensity of C3, C4, C1q and IgG1-4 in renal tissue.

**Figure 2:**
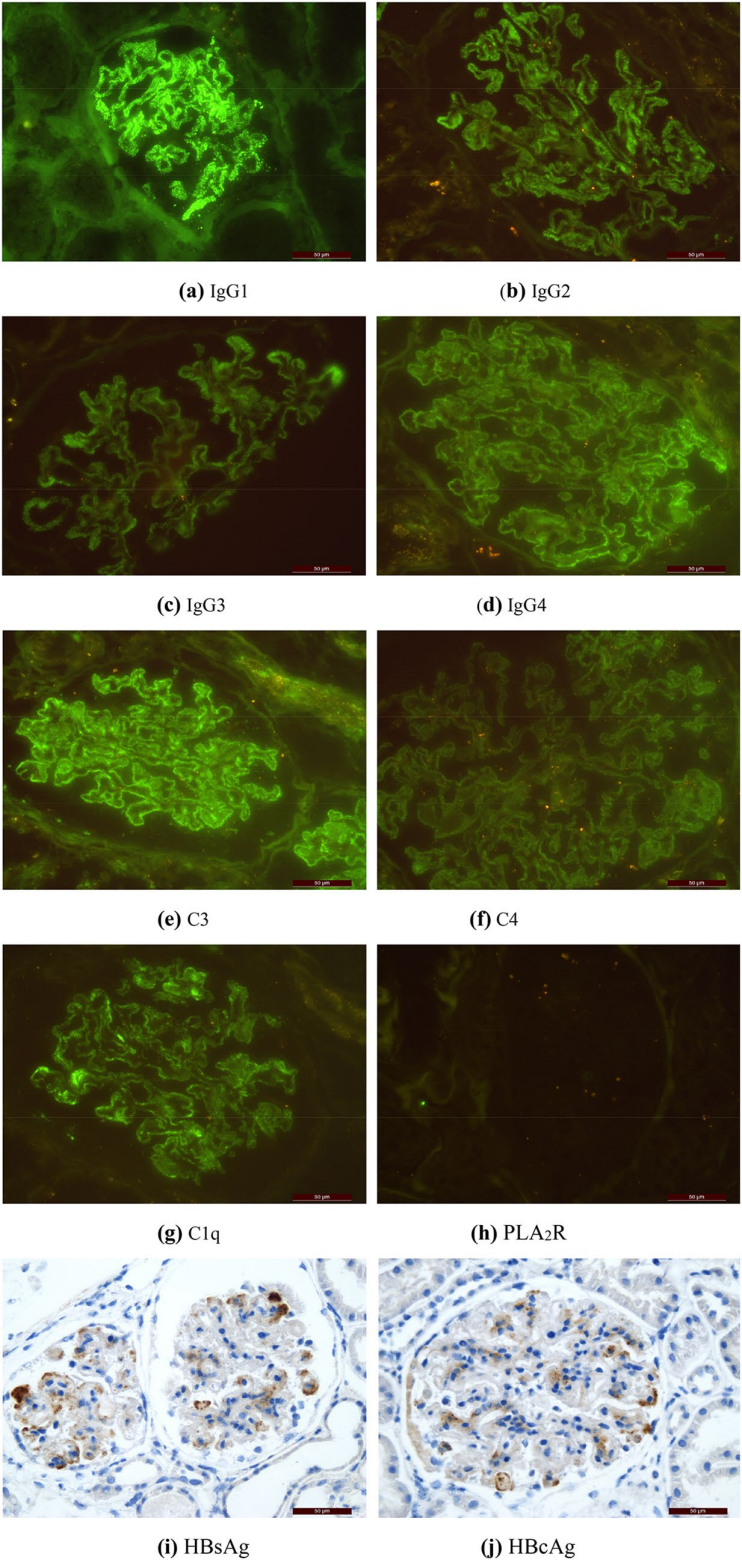
HBV-MN group: (**a**–**h**) showed the average deposition intensity of IgG1-4, C3, C4, C1q, PLA_2_R detected by immunofluorescence method, and (**i**) and (**j**) showed the detection of HBsAg and HBcAg by immunohistochemical staining respectively. (Original magnification × 400 for all above).

**Figure 3:**
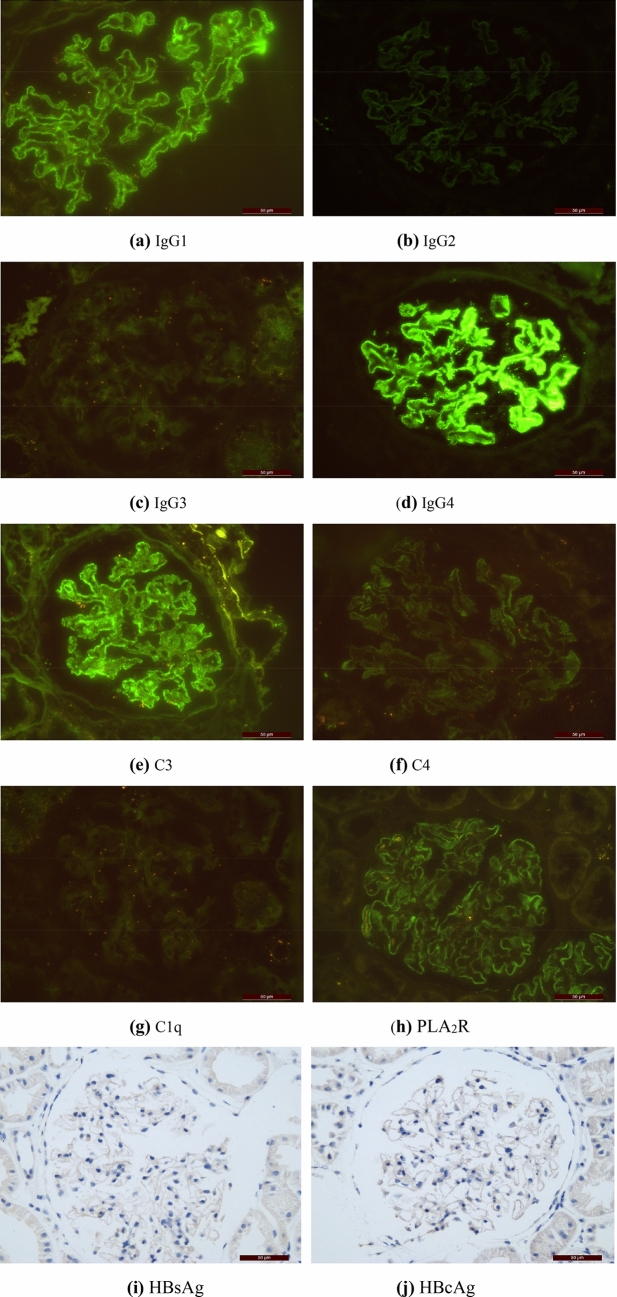
IMN group: (**a**–**h**) showed the average deposition intensity of IgG1-4, C3, C4, C1q, PLA_2_R detected by immunofluorescence method, and (**i**) and (**j**) showed the detection of HBsAg and HBcAg by immunohistochemical staining respectively. (Original magnification × 400 for all above).

Among the 50 patients with HBV-MN, 37 reported IgG1 ~ 4 deposition. IgG1, IgG2, IgG3 and IgG4 were deposited in 36 cases (97.30%), 26 cases (70.27%), 21 cases (56.76%) and 22 cases (59.46%), respectively. 56 cases of IMN group with HBV infection reported IgG1 ~ 4 deposition. IgG1, IgG2, IgG3 and IgG4 deposited in 47 cases (83.93%), 37 cases (66.07%), 28 cases (50%) and 50 cases (89.29%), respectively. Of the 50 HBV-MN patients, 48 patients (96.00%) were found C3 deposition, 33 patients (71.74%) were found C4 deposition, 39 patients (79.59%) were found C1q deposition. Of the 56 IMN patients, 48 patients (85.71%) had C3 deposition, 34 patients (61.82%) had C4 deposition, 29 patients (51.79%) were found C1q deposition (Fig. 1, Table 3). Figure 2 and Fig. 3 show the average deposition intensity of IgG1-4, C3, C4, C1q and PLA_2_R detected by immunofluorescence and the deposition of HBsAg and HBcAg detected by immunohistochemical staining in the HBV-MN and IMN groups, respectively.

**Table 3:** The deposition intensity of C3, C4, C1q, IgG1-4, and PLA_2_R in renal tissue.

Group	IgG	IgG1	IgG2	IgG3	IgG4	C3	C4	C1q	PLA_2_R
Pts									
HBV-MN	50	36	26	21	22	48	33	39	5
IMN	56	47	37	28	50	48	34	29	12
*χ*2	3.711	8.796	0.405	0.345	4.994	1.467	3.514	11.471	2.562
*P*	0.054	0.003	0.525	0.557	0.025	0.226	0.061	0.001	0.109

### Comparison of IgG deposits intensity intra-group

We also compared the deposition intensity of IgG between intra-group. A difference was found in the serum levels of IgG1/IgG2 and IgG1/IgG3 in the HBV-MN group (*P* < 0.0083). There was no difference between IgG1/IgG2, IgG1/IgG3, IgG1/IgG4, IgG2/IgG3, and IgG3 /IgG4 in the IMN complicated with HBV infection group (Tables 4, 5).

**Table 4:** The deposition intensity of IgG deposits intensity between the two groups.

Group	Mean rank of IgG subclass				*χ*2	*P*
	IgG1	IgG2	IgG3	IgG4		
HBV-MN	100.68	59.28	55.96	82.08	28.187	0.000
IMN	124.11	83.28	78.14	164.47	68.219	0.000

**Table 5:** The deposition intensity of IgG deposits intensity intra-group.

Group	*P* value of comparison of 4 IgG subclass in different groups					
	IgG1 vs IgG2	IgG1 vs IgG3	IgG1 vs IgG4	IgG2 vs IgG3	IgG2 vs IgG4	IgG3 vs IgG4
HBV-MN	0.000	0.000	0.485	0.514	0.073	0.038
IMN	0.000	0.000	0.000	0.382	0.000	0.000

In conclusion, compared with the IMN complicated with HBV infection group, stronger IgG1 and C1q and weaker IgG4 staining were found in HBV-MN group renal tissues. The deposition IgG1, C1q and IgG4 showed a statistically significant difference between the two groups (*P* = 0.003, 0.025, and 0.001, respectively).

### Indicators of clinical and biochemical in HBV-MN

Fluorescence quantitative polymerase chain reaction was used to detect HBV DNA replication and its replication level in serum of patients with HBV-MN (range of 10^2^–10^7^ copies/mL). Then the patients were divided into 2 groups according to the replication level, namely the normal group (DNA < 5 × 10^2^ copies/mL) and the virus replication group (DNA ≥ 5 × 10^2^ copies mL). There were 50 patients with HBV-MN, including 14 patients in the normal group, 36 patients in the virus replication group. The ALT of patients in the normal group was significantly lower than that in another group (*P* < 0.05), but there were no significant differences in ALB, AST, SCR, BUN, UA, T-CHO and 24 h-TP (Table 6).

**Table 6:** Biochemical indexes in different groups (according to the serum replication level of HBV DNA).

Clinical index	DNA ≥ 5 × 10^2^ copies/mL	DNA < 5 × 10^2^ copies/mL	*P*
BUN (mmol/L)	5.18 ± 0.130	4.51 ± 0.389	0.075
SCR (µmol/L)	72.69 ± 26.451	66.21 ± 5.360	0.482
UA (µmol/L)	350.19 ± 72.672	306.14 ± 20.556	0.116
ALT (U/L)	47.83 ± 5.592	19.50 ± 2.974	0.002
AST (U/L)	45.58 ± 4.445	30.00 ± 5.463	0.057
ALB (g/L)	24.32 ± 3.067	24.47 ± 1.681	0.936
T-CHO (mmol/L)	7.31 ± 0.577	6.99 ± 0.652	0.863
24 h-TP (g)	5.87 ± 0.752	6.50 ± 0.908	0.489
C3 (g/L)	0.99 ± 0.006	1.07 ± 0.045	0.071
C4 (g/L)	0.23 ± 0.025	0.24 ± 0.022	0.648

### Survival analysis

**Figure 4:**
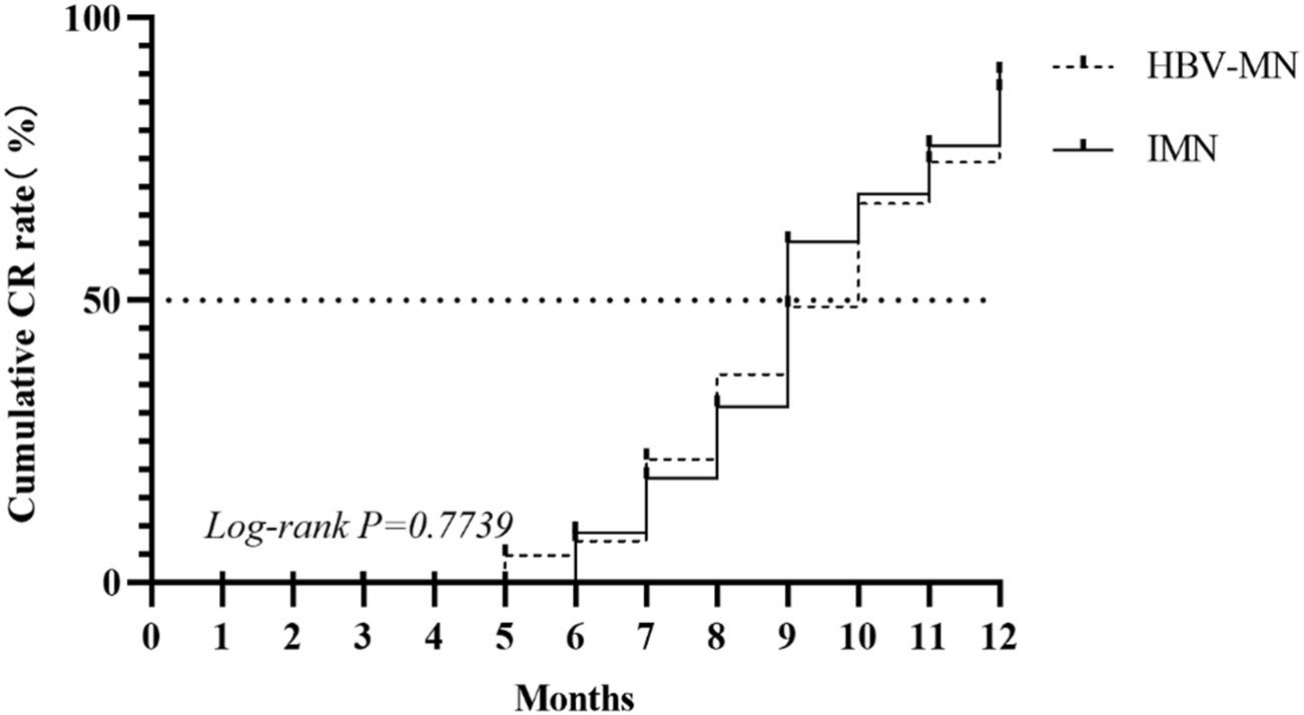
Survival curve of CR in patients in HBV-MN compared with curve of patients in IMN.

After the diagnosis is clear, the antiviral therapy of entecavir and the use of hormones and / or immunosuppressant tacrolimus (TAC) are decided according to the HBV DNA replication of the patient. The patients were followed up for 1 year to observe the remission of nephropathy. The primary outcome variables were the number of patients who reached complete remission (CR). CR was defined as < 0.3 g/d proteinuria, meanwhile serum creatinine and albumin levels were normal. Kaplan–Meier analysis was used for survival analysis, and log-rank analysis was used for comparison of survival curves (*P* > 0.01) (Fig. 4).

## Discussion

Combes et al. published the first report on the association between chronic HBV infection and kidney disease in 1971^4^. Histologically, several lesions have been identified. These include MN, membranoproliferative glomerulonephritis, immunoglobulin (Ig) A nephropathy, focal segmental glomerulosclerosis^5^. Patients may have one distinct histologic lesion or have overlapping features in a single kidney biopsy. The predominance of a particular lesion may depend upon the qualitative and quantitative differences in the immune complexes formed^6^. Hepatitis B virus associated glomerulonephritis (HBV-GN) is a kind of immune complex-mediated glomerulonephritis, but its specific pathogenesis is not clear. The pathogenetic mechanisms by which individuals develop nephropathy are probably dependent on interactions between viral, host and environmental factors.

Comparing the demographic, clinical and pathological features of HBV-MN and IMN, it was found that there were significant differences between them. There were significant differences in average age and serum complement. These differences can be attributed to the pathological changes of the disease: IgG deposits, complement deposition and so on. Some studies have shown that HBV-MN is mainly seen in young men, while IMN is more common in middle-aged men^5,7,8^. In this study, both groups were mainly male, and the age of onset in patients with HBV-MN was significantly lower than that in patients with IMN. Therefore, the possibility of HBV-associated membranous nephropathy should be considered in young people with atypical membranous nephropathy. Hepatitis B virus markers in serum and renal tissue should be detected during a renal biopsy, especially in patients with a history of hepatitis B.

Most HBV-GN patients have a history of chronic HBV infection and serum HBsAg is positive. However, in our study, 6 patients with seronegative HBV markers were observed in the HBV-MN group. Recent studies have also found that some HBV-GN patients are serum HBsAg-negative^9–11^. We can speculate that, on the one hand, the recovery of glomerulonephritis lags behind the regression of HBV infection. On the other hand, negative HBsAg may represent occult hepatitis B virus infection (OBI) in HBV-GN^12^. OBI may occur after clinical complete recovery of acute self-limited hepatitis, HBsAg serum clearance does not necessarily mean HBV eradication^13,14^. The extremely low HBV DNA viral load, HBsAg variation, HBV subtype variation and immune escape strain in serum contribute toward the separation of HBV markers in serum and renal tissue^11^. In some patients, HBV markers can be observed in renal tissue, but they are negative in serum. So, negative HBV markers in serum cannot rule out HBV-MN, and detection of HBV markers in renal tissue is very important for the diagnosis of HBV-MN.

HBV-related nephropathy affects adults in different regions with various clinical manifestations. In East Asia, most HBV-GN patients presented with nephrotic syndrome and some showed mild to moderate proteinuria with hematuria, and some still developed progressive renal failure^15^. Our study shows that nephrotic syndrome is the main clinical manifestation of the two groups, but from the level of serum ALB and 24 h-TP, HBV-MN and IMN can’t be distinguished in patients with HBV infection. There was no significant difference in hypertension, renal insufficiency, liver insufficiency and liver cirrhosis between the two groups. From the results of laboratory examination, the levels of serum complement C3 and C4 in the HBV-MN group were significantly lower than those in the IMN group. Complement was synthesized in the liver, and serum complement decreased significantly when the liver function was damaged^16–18^. There was no significant difference in liver insufficiency and liver cirrhosis between the two groups, which may be related to circulating immune complex activated complement^6,19,20^. However, there was no significant statistical difference in the levels of BUN, SCR, UA, ALT, AST and T-CHO. There was no significant difference in serum PLA_2_R positive rate between the two groups. In addition, one patient in the HBV group was positive for PLA_2_R in renal tissue but negative in serum antibody, which may be because serum PLA_2_R may change with the development of the disease, while PLA_2_R in renal tissue is relatively stable^21^.

It is generally believed that virus-associated glomerulonephritis occurs only in the context of active replicative virus infection and is resolved with the eradication of the virus status^3^. There is also a view that our HBV markers may not be detected in serum after virus clearance. However, the renal pathological changes caused by previous HBV infection are irreversible^7^. Some experimental studies have shown that purified HBV can directly promote the proliferation of human mesangial cells (HMC) and the expression of type IV collagen and fibronectin^22^, which proved that the pathogenesis of hepatitis B virus glomerular disease is directly related to the virus. High levels of HBV DNA replication also increase the possibility of kidney-associated antigen deposition, forming immune complexes, leading to the activation of various inflammatory cells and cytokines^23^. Many clinical studies have shown that with the increase of serum HBV DNA level, the clinical manifestations and other biochemical indexes of the patients also changed. However, in our study, we only observed that when the replication level of HBV DNA was less than 5 × 10^2^ copies/mL, the level of ALT decreased significantly.

Renal biopsy is still the gold standard for the diagnosis of HBV-MN. There were significant differences in pathological changes between HBV-MN and IMN. Recent studies have shown that the detection of PLA_2_R antigen and its dominant IgG4 autoantibodies in the glomerular deposition in patients with MN are helpful to distinguish IMN from secondary MN (SMN)^24^. IgG4 is usually the most significant deposition of IgG subclass in IMN. On the contrary, in SMN, the deposition of IgG1, IgG2 and IgG3 exceeds that of IgG4^25^. At the same time, some studies have shown that renal PLA_2_R and serum PLA_2_R are not only expressed in IMN, but also expressed in HBV-MN patients^26^. In our study, 5 cases (10%) of HBV-MN patients were positive for PLA_2_R. This also suggests that we should not blindly diagnose IMN when only PLA_2_R is positive, but also exclude the possibility of SMN. Our study found that compared with the IMN group with HBV infection, the renal tissue IgG1 and C1q were stronger and the staining was weaker in the HBV-MN group. It is suggested that IgG subclass and C1q play an important role in differentiating the two, in which the stronger deposition of IgG1 and C1q in renal tissue is more inclined to diagnose HBV-MN, and the stronger deposition of IgG4 is more inclined to diagnose IMN. Unfortunately, the HBeAg deposition was not listed in the renal biopsy due to technical limitations. Studies showed that the capillary wall lesion of HBV-associated glomerulopathy may be associated with the deposition of HBV-associated antigens, especially HBeAg and IgG antibodies, on the capillary wall of glomerulopathy^27–30^. The principles for the treatment of HBV-associated glomerulonephritis are: (1) reducing proteinuria; (2) preventing and treating recurrence; (3) protecting renal function and delaying the progression of renal disease. Antiviral therapy is the main treatment of HBV-GN. Even HBV-GN with negative serum HBsAg could not achieve satisfactory results with immunosuppressants alone. Combined antiviral therapy is effective and may be necessary^9^. Entecavir is the first choice because of its antiviral effect and low drug resistance tendency^31^. However, antiviral therapy alone has some limitations, such as HBV mutation, low remission rate and long average remission time^32^. A recent study has shown that TAC may inhibit the entry of HBV into hepatocytes by targeting the candidate HBV receptor NTCP. Studies have shown that TAC can not only reduce proteinuria, but also inhibit HBV DNA replication, and confirmed the beneficial effects of TAC combined with entecavir therapy in Chinese adult HBV-GN patients, such as significant relief of proteinuria. This combination does not increase HBV DNA replication or acute renal function deterioration^32^. The use of corticosteroids is still controversial because patients receiving corticosteroids are temporary, incomplete or unbeneficial, so they should be very cautious when using corticosteroids. After the diagnosis was confirmed, we decided on the antiviral therapy of entecavir and the use of hormone and / or immunosuppressant TAC according to the HBV DNA replication of the patient. We followed up patients in the two groups for 1 year. Unfortunately, there was no significant difference in protein remission rate between the two groups. We analyzed the reasons for this, which may be related to the small sample size, short follow-up time and high proportion of deleted data. Nevertheless, a clear diagnosis is important for treatment planning.

The shortcomings of this study are as follows: First, due to the limited selection conditions, the recruited subjects in this study were only from a single center of the First Affiliated Hospital of Zhengzhou University, which limited the sample size. But HBV genotype has regional characteristics. Second, because the pathological diagnosis technology of early renal biopsy in our hospital is not fully mature, some detection items are missing in the pathological report. Third, renal biopsy is an invasive operation with relatively few samples, which may lead to missed diagnosis due to sampling.

In conclusion, serological examination of negative HBV antigen could not rule out HBV-MN. Serum C3 and C4 were lower in patients with HBV-MN than in patients with IMN combined with HBV infection. In a renal biopsy, complement C3 and IgG deposition were dominant in both groups. The deposition intensity of complement C3 in the HBV-MN group was higher than that in the IMN with HBV infection group. The immunofluorescence IgG subtypes in the HBV-MN group were mainly IgG1 deposition, while those in the IMN with HBV infection group were mainly IgG4 deposition. When HBV-associated antigen and PLA_2_R are present in renal tissue, lower level of serum C3 and C4, high intensity of renal C1q and IgG1 is more supportive of HBV-MN. However, the positive expression of PLA_2_R in serum and / or renal tissue in differentiating HBV from IMN complicated with HBV infection remains to be discussed. After 1-year follow-up, there was no difference in the remission rate of proteinuria between the two groups. After a clear diagnosis, a standardized treatment regimen may improve the prognosis of patients.

## Methods

### Criteria of the patients

Patients who had been treated in The First Affiliated Hospital of Zhengzhou University from January 2013 to December 2018 were selected. 50 patients (38 males and 12 females, male-to-female ratio of 3.17:1) who underwent renal biopsies and were diagnosed with HBV-MN were enrolled in this study. 56 IMN patients with HBV infection (38 males and 18 females, male-to-female ratio of 2.11:1), diagnosed during the same period were included as controls. In this study, the diagnostic criteria for HBV-MN are as follows: (1) positive serum HBV antigen; (2) membranous nephropathy, excluding secondary membranous nephropathy such as lupus nephritis, and (3) HBV antigen (HBsAg and / or HBcAg) was found in renal tissue sections.

The pathological diagnosis of HBV-MN is mainly referred to membranous nephropathy. The histopathological features of HBV-MN are as follows: In addition to diffuse glomerular basement membrane thickening and napped, the thickened basement membrane can also be chained and accompanied by obvious mesangial hyperplasia under light microscope. Immunofluorescence examination showed that in addition to the granular deposition of IgG and C3 along the capillary wall, there were also IgM, IgA and C1q deposits, even similar to "full bright". Electron microscopic examination showed that large electron densities were distributed in multiple locations, including subepithelial, basement membrane, subcutaneous, and mesangial regions.

Patients who met criteria (1) + (2) + (3) or (2) + (3) were diagnosed with HBV-MN^12,26^. Diagnosis of IMN by biopsy was consistent with the Ehrenreich standards^33^. Parameters including blood routine, urine routine and plasma levels of ALB, SCR, BUN, UA, T-CHO, TG, C3, C4, ALT, and AST, 24 h-TP, PLA_2_R and HBV related markers during the hospitalization and outpatient follow-up study period were collected for all the patients. This study was approved by the Ethics Committee of the First Affiliated Hospital of Zhengzhou University (Henan, China, No.2019-KY-015). Statement: All methods were performed in accordance with the relevant guidelines and regulations.

### Pathological classification and diagnosis criteria

The diagnosis of nephropathy was confirmed by pathology. Renal biopsy procedures include light microscopy, immunofluorescence, immunohistochemistry, and electron microscopy for the pathological diagnosis of the kidney. Renal tissue samples were routinely processed. Routine light microscopic examinations included hematoxylin–eosin staining (HE), Masson staining, periodic acid-silver metheramine (PASM) and periodic acidSchiff stain (PAS). For direct immunofluorescence, the frozen sections of fresh tissue specimens were stained with antisera against human IgG1, IgG2, IgG3, IgG4, C3, C4 and C1q, and scaled from 0 to 4 + . Indirect immunofluorescence staining was used to detect the expression of PLA_2_R in renal tissue. The expression of HBsAg and HBcAg in renal tissue was detected by immunohistochemical staining. Electron microscopy was used to identify dense deposits.

### Statistical analysis

SPSS version 26.0 software was used for the statistical analysis. Results from normally distributed variables were expressed as means ± standard deviation (SD), while t-test was performed to compare differences between groups. Non–normal variables were expressed as medians and interquartile range (IQR), and differences between groups were compared by Mann–Whitney U or Kruskal Wallis test. Chi-square test was used for the comparison of qualitative data. Kaplan–Meier analysis was used for survival analysis, and Log-rank analysis was used for comparison of survival curves. The difference of *P* < 0.05 was considered to have statistical significance.

### Statement

The informed consent was obtained from all subjects (above 18 years old) and the informed consent was also obtained from parent and/or legal guardian of participants less than 18 years old.
